# Biogenic Volatile Organic Compounds and Protein Expressions of *Chamaecyparis formosensis* and *Chamaecyparis obtusa* var. *formosana* Leaves under Different Light Intensities and Temperatures

**DOI:** 10.3390/plants11121535

**Published:** 2022-06-08

**Authors:** Ying-Ju Chen, Ya-Lun Huang, Yu-Han Chen, Shang-Tzen Chang, Ting-Feng Yeh

**Affiliations:** 1 School of Forestry and Resource Conservation, National Taiwan University, Taipei 10617, Taiwan; yingju@tfri.gov.tw (Y.-J.C.); yalunhuang@ntu.edu.tw (Y.-L.H.); f97625037@gmail.com (Y.-H.C.); 2 Division of Forest Chemistry, Taiwan Forestry Research Institute, Taipei 10070, Taiwan

**Keywords:** biogenic volatile organic compound, cypress leaf, light intensity, protein expression, temperature

## Abstract

Both *Chamaecyparis formosensis* and *C. obtusa* var. *formosana* are representative cypresses of high economic value in Taiwan, the southernmost subtropical region where cypresses are found. Both species show differences of their habitats. To find out the effects of environmental factors on the CO_2_ assimilation rate and the biogenic volatile organic compound (BVOC) emission of both species, saplings from both species were grown under different light intensity and temperature regimes. The results indicated that the net CO_2_ assimilation rates and total BVOC emission rates of both species increased with increasing light intensity. *C. formosensis* showed a higher magnitude of change, but *C. obtusa* var. *formosana* had considerably increased sesquiterpenoid and diterpenoid emission in BVOC under high light intensity. Both species grown under higher temperatures had significantly lower BVOC emission rates. Proteomic analyses revealed that compared to *C. formosensis* saplings, *C. obtusa* var. *formosana* saplings had less differentially expressed proteins in terms of protein species and fold changes in response to the growth conditions. These proteins participated mainly in photosynthesis, carbon metabolism, amino acid and protein processing, signal transduction, and stress mechanisms. These proteins might be the major regulatory factors affecting BVOC emission of these two species under different environments.

## 1. Introduction

Both *Chamaecyparis formosensis* and *C. obtusa* var. *formosana* are considered representative cypresses of high economic value in Taiwan. They are very popular in construction, furniture, interior decoration, and the fragrance industry in aromatherapy. Globally, cypress forests are found in North America, Japan, and Taiwan. Among these places, Taiwan is the southernmost region and the only subtropical region where cypresses are found [1]. Field studies showed slight differences in their habitats. *C. formosensis* grows mainly in valleys [2] whereas *C. obtusa* var. *formosana* grows mainly on ridges [3]. In addition, *C. formosensis* saplings are phototropic and require sufficient light for growth, while *C. obtusa* var. *formosana* saplings prefer to grow in the understory. The different habitat requirements of the two species may be due to natural selection or adaptation [4]. Presently, the reason behind the difference in distribution of the two species remains inconclusive. Their responses to primary metabolic systems have been examined [5]; whether secondary metabolic systems participate in regulating the environmental adaptation capacity of plants as well as their possible roles merit further investigation.

Volatile organic compounds (VOCs) emitted via biological metabolisms are known as biogenic VOCs (BVOCs). Plants produce diverse BVOCs through precise and complex synthesis reactions. Examples of BVOCs include oxylipins, green leaf volatiles, terpenoids, and various carotenoid derivatives, indoles, and phenolics [6]. Among these compounds, volatile terpenoids account for more than half of BVOCs emitted [7]. From a plant energy metabolism perspective, terpenoid emission consumes energy and slows down growth rate [8], which are undoubtedly undesirable for plants. Therefore, biologists deduce that BVOC emission by plants must serve some physiological or ecological functions. Many studies found that plant synthesis or emission of terpenoids is a protective mechanism against temperature [9], oxidative damage [10], or drought stress [11]. Moreover, terpenoids inhibit pathogenic bacteria and herbivorous insects, and attract pollinators [12,13]. These help plants overcome stress, resist invasion, and adapt to its growth environment.

Gene expression and regulation ensure that proteins are expressed at specific times and sites and at suitable levels to carry out biological functions. Through proteomics analysis, scientists are able to confirm the differences in gene products (proteins) for a better understanding of their possible functions and their regulatory mechanisms in various physiological and biochemical pathways [14,15]. Few publications have used a proteomic approach to address the variations of cypress’s BVOCs caused by environmental factors. In this study, a phytotron-controlled environment was employed to examine the effects of environmental factors on photosynthesis, chemical composition, and content of BVOCs emitted from Taiwan cypresses under different environmental conditions. Proteomic analyses of the saplings under different environments were performed to shed light on whether environmental factors directly affect the expression of key enzymes in these metabolic pathways. Findings of this study would help identify potential regulatory proteins and determine their functions to understand the regulatory models by which plants respond to certain environmental changes, and this is clearly a prerequisite for designing strategies to improve the competitiveness of plants grown under unfavorable conditions brought by the ongoing climatic change.

## 2. Results and Discussion

### 2.1. Net CO_2_ Assimilation Rate of C. formosensis and C. obtusa var. formosana Saplings in Controlled Environments

**Figure 1:**
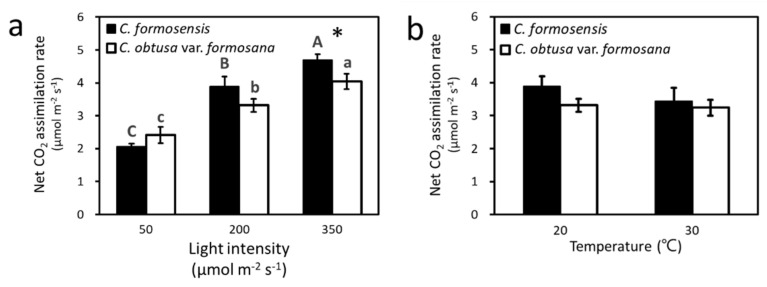
Net CO_2_ assimilation rates of *C. formosensis* and *C. obtusa* var. *formosana* saplings grown at (**a**) different light intensities under 20 °C, and (**b**) different temperatures under 200 μmol m^−2^ s^−1^ light intensity. Bars (mean ± s.e., *n* = 9) with different letters denote significant difference in means among the three light intensities in each species. Bars with a star indicate significant difference in means between two species at that light intensity (*, *p* < 0.05).

Nine saplings with similar genetic backgrounds and BVOCs were selected (Supplementary Methods) and grown in a phytotron-controlled environment under different temperature and light intensity conditions for two weeks. At 20 °C, the net CO_2_ assimilation rates of *C. formosensis* and *C. obtusa* var. *formosana* at the three light intensities or photosynthetic photon flux densities (PPFDs) were significantly different (*C. formosensis*: *F*_2,16_ = 66.95, *p* < 0.001 and *C. obtusa* var. *formosana*: *F*_2,16_ = 24.33, *p* < 0.001) (Figure 1a). However, both species showed the same trend of highest rate at PPFD = 350 μmol m^−2^ s^−1^, lower at PPFD = 200 μmol m^−2^ s^−1^, and lowest at PPFD = 50 μmol m^−2^ s^−1^ (Figure 1a). Generally speaking, plants’ net CO_2_ assimilation rates increase with increasing light intensity lower than the light saturation points of plants [16]. In our preliminary measurement, the light saturation points of these two species were higher than the three light intensities tested; therefore, the net CO_2_ assimilation rates of both species increased with light intensity. Comparing the net CO_2_ assimilation rates of both species showed significant difference only under high light intensity (PPFD = 350 μmol m^−2^ s^−1^, Figure 1a) (*F*_1,16_ = 4.86, *p* < 0.05). This result implied that *C. formosensis* is more light-demanding than *C. obtusa* var. *formosana*, which is consistent with the differences in their dominant habitat [2,3,17] and leaf growth dynamics [5].

As for the effects of temperature on the net CO_2_ assimilation rates of *C. formosensis* and *C. obtusa* var. *formosana* at PPFD = 200 μmol m^−2^ s^−1^ (Figure 1b), the two species showed no difference in net CO_2_ assimilation rates between 20 °C and 30 °C (*C. formosensis*: *F*_1,8_ = 1.79, *p* = 0.2 and *C. obtusa* var. *formosana*: *F*_1,8_ = 0.1, *p* = 0.8). In general, plants’ net CO_2_ assimilation rates decreases with increasing temperature higher than its optimal growing temperature [18,19]. The optimal temperatures of these two species were below 30 °C [20]. However, in this study, the net CO_2_ assimilation rates of both species at 30 °C were not significantly lower than those at 20 °C. Our previous study also found that the net CO_2_ assimilation rates of these two species at 30 °C were not lower than those at 20 °C when sapling grown in growth chamber only for one month [20]. Therefore, the insignificant reduction in the net CO_2_ assimilation rates of these two species observed at 30 °C in this study might be due to the short-controlled treatment period.

### 2.2. BVOC Emissions from C. formosensis and C. obtusa var. formosana Saplings in Controlled Environments

**Figure 2:**
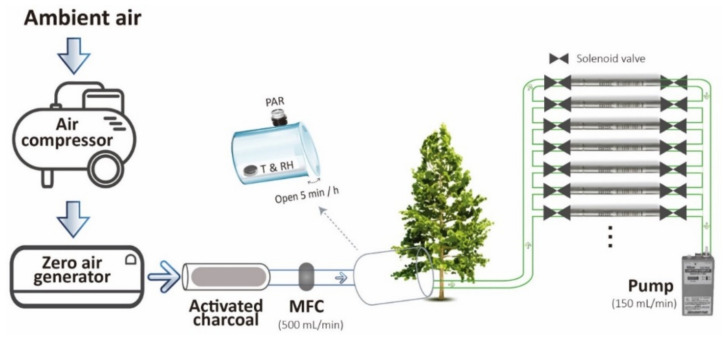
Scheme of sampling devices for collecting the BVOCs emitted from leaves. MFC, mass flow controller; PAR, photosynthetically active radiation; T, temperature; RH, relative humidity.

**Figure 3:**
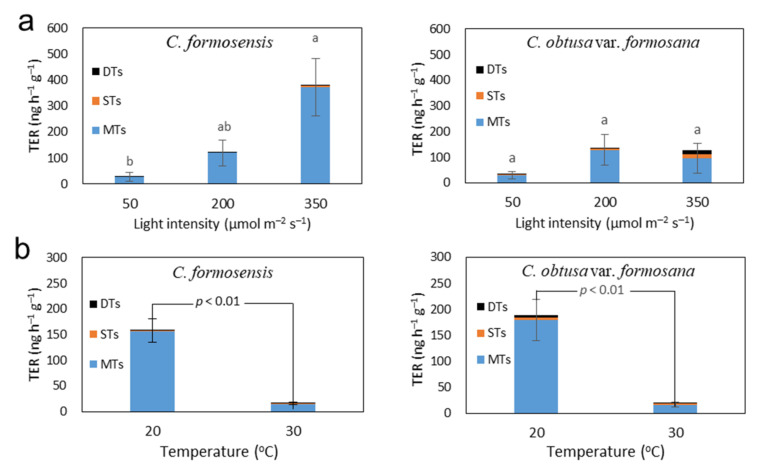
Total BVOC emission rates (TERs) of *C. formosensis* and *C. obtusa* var. *formosana* saplings grown at (**a**) different light intensities under 20 °C, and (**b**) different temperatures under 200 μmol m^−2^ s^−1^ light intensity. MTs, monoterpenoids; DTs, diterpenoids; STs, sesquiterpenoids. Bars (mean ± s.e., *n* = 3) with different letters denote significant difference in means among the three light intensities at *p* < 0.05 level by Scheffe’s test.

To understand the relationships of BVOC emission rates of *C. formosensis* and *C. obtusa* var. *formosana* with light intensity and temperature, the total BVOC emission rates of saplings at different light intensities and temperatures were measured. The sample devices for collecting the BVOCs from sapling leaves are shown in Figure 2. Results indicated an increase in BVOC emission rate of *C. formosensis* saplings with increasing light intensity (Figure 3a). The total BVOC emission rates were 27.2 ± 17.5 ng h^−1^ g^−1^ at PPFD 50, < 121.1 ± 40.3 ng h^−1^ g^−1^ at PPFD 200, and < 378.5 ± 40.3 ng h^−1^ g^−1^ at PPFD 350. Similarly, the emission rate of *C. obtusa* var. *formosana* also increased with light intensity (Figure 3a). The total BVOC emission rates were 33.7 ± 13.5 ng h^−1^ g^−1^ at PPFD 50, 136.4 ± 59.5 ng h^−1^ g^−1^ at PPFD 200, and 127.5 ± 58.1 ng h^−1^ g^−1^ at PPFD 350.

On the other hand, the BVOC emission rates of sapling leaves of both *C. formosensis* and *C. obtusa* var. *formosana* at 30 °C were significantly less than those at 20 °C (Figure 3b). At 20 °C, the total BVOC emission rates were 159.1 ± 23.1 ng h^−1^ g^−1^ (*C. formosensis*) and 188.9 ± 39.7 ng h^−1^ g^−1^ (*C. obtusa* var. *formosana*); when the temperature rose to 30 °C, the emission rates decreased to 17.2 ± 2.7 ng h^−1^ g^−1^ (*C. formosensis*) and 21.4 ± 4.8 ng h^−1^ g^−1^ (*C. obtusa* var. *formosana*). The results revealed that BVOC emission rates in saplings of both species tended to correlate positively with light intensities but negatively with temperatures, especially in *C. formosensis*. Such correlations are consistent with the results from outdoor experiments obtained by Chen et al. (2019), which indicated a greater effect of light intensity on BVOC emission rates of *C. formosensis* saplings than that of temperature [21].

In research on release of BVOCs from plants, isoprene has been the most thoroughly studied compound. According to the theoretical basis of the G93 model, which is currently the mostly widely applied model for basal emission rate estimation, the release of stored isoprenoids from the storage area was mainly through diffusion, and was not related to the physiological process. By contrast, the emission of non-stored isoprene was restrained by the biosynthesis rate. The extent of effects of light intensity on isoprene emission rate reflected the dependence of isoprene biosynthesis on primary substrate products generated through photosynthesis, whereas the variations in isoprene emission rate with temperature reflected the extent of effects of temperature on terpene synthases activity in BVOC biosynthetic pathways [22]. Although the leaves of both *C. formosensis* and *C. obtusa* var. *formosana* were found to contain resin ducts pertaining to the release of stored terpenoids, the resin ducts in sapling leaves of *C. formosensis* were less obvious compared with those in the adult tree leaves, and the terpenoid content of sapling leaves was significantly lower than that of adult trees [21]. Thus, this study speculated that BVOC emission rates of saplings were more susceptible to light intensity than temperature, which was probably attributed to the substrate requirement for the biosynthesis of photosynthesis-generated terpenoids.

Many researchers suggested that the response of isoprenoid emission rate to temperature was probably influenced by the activity of isoprene synthase and the substrate limitation [22,23]. When the temperature exceeds the optimal temperature for photosynthesis, the activity of rate-limiting enzymes in the biosynthetic pathways of BVOC chloroplasts might be constrained. Thus, the emission of isoprenoids might be restrained by synthetic substrates. At higher temperatures, the emission of BVOCs was more sensitive to the effect of light intensity, and reached peak emission more rapidly [22]. Therefore, the lower BVOC emission rates at higher temperatures might result from the lower enzyme activity caused by high temperature stress, and also from the rapid exhaustion of the substrate at higher temperatures.

Although total BVOC emission rates and the net CO_2_ assimilation rates of *C. formosensis* saplings tended to rise with increasing light intensities, the total BVOC emission rate of *C. obtusa* var. *formosana* saplings seemed to decreased slightly (yet with insignificant difference; *p* > 0.05) at high light intensity (PPFD = 350 μmol m^−2^ s^−1^). However, the emissions of sesquiterpenoids (STs) and diterpenoids (DTs) of *C. obtusa* var. *formosana* at high light intensity (PPFD = 350 μmol m^−2^ s^−1^) increased compared with those at low (PPFD = 50 μmol m^−2^ s^−1^) and medium (PPFD = 200 μmol m^−2^ s^−1^) light intensities (Figure 3a). According to previous studies, high light intensities cause leaf temperature to rise, resulting in thermal stress as well as photo-oxidative stress within the chloroplasts [24,25], which further restrain the photosynthesis and biosynthetic enzymes of BVOCs. These stresses induce an increase in emission of sesquiterpenes. Thus, the present findings revealed that at a higher temperature of 30 °C or high light intensity of PPFD = 350 μmol m^−2^ s^−1^, both *C. formosensis* and *C. obtusa* var. *formosana* might be under stress. Under higher temperature, BVOC emission rates of both species decreased significantly. However, under high light intensity, BVOC emission rates of *C. formosensis* continued to increase without significant change in its BVOC composition. To determine the factors affecting emission behavior of *C. formosensis* and *C. obtusa* var. *formosana* as well as their different physiological regulation mechanisms under stress, variations in protein expression of saplings’ leaves at different light intensities and temperatures were analyzed.

### 2.3. Differential Proteomic Analysis of Chamaecyparis Leaves under Different Light Intensities and Temperatures

The extracted proteins from both *C. formosensis* and *C. obtusa* var. *formosana* leaf samples were grouped and labeled with the difference gel electrophoresis kit (DIGE-Kit) according to Table S1. 2D-DIGE proteomic analysis coupled with LC-MS was carried out to identify differential protein expressions under different light intensities (PPFD 200/PPFD 50; PPFD 350/PPFD 200) and temperatures (30 °C/20 °C). The protein expressions with ratio > 1.00 or < 1.00 and with *p* < 0.05 were selected as differential protein spots. In *C. formosensis* and *C. obtusa* var. *formosana*, 46 and 15 proteins met these criteria, respectively. Among the differential proteins of *C. formosensis*, 21 were upregulated while 20 were downregulated with only increasing light intensity; 9 were upregulated while 20 were downregulated with only increasing temperature (Figure S1). Among the differential proteins of *C. obtusa* var. *formosana*, 6 were upregulated while 9 were downregulated with only increasing light intensity; 2 were upregulated while 4 were downregulated with only increasing temperature (Figure S2).

### 2.4. Protein Identification and Functional Analysis

**Figure 4:**
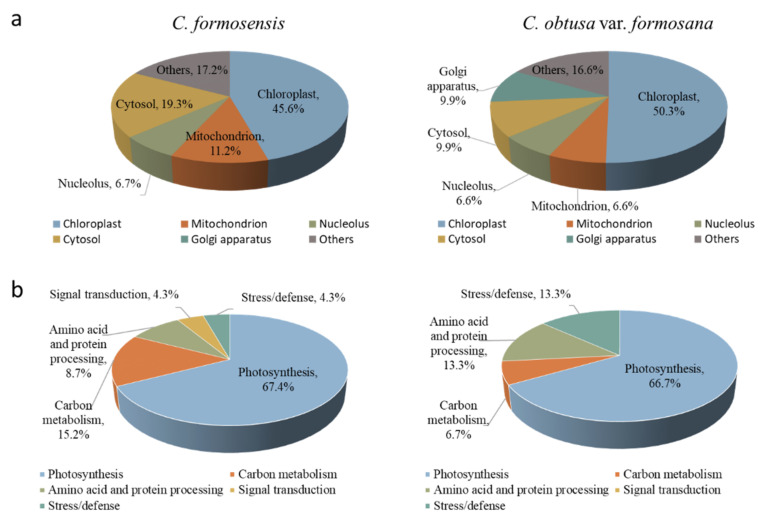
Subcellular localizations (**a**) and functional distribution (**b**) of identified proteins of *C. formosensis* and *C. obtusa* var. *formosana*.

These 61 protein spots with significantly different expressions were further analyzed by LC-MS, and their sequence information was obtained from the MASCOT database search and related analyses. Gene ontology analysis through Blast2GO revealed that 45.6% and 50.3% of differentially expressed proteins of *C. formosensis* and *C. obtusa* var. *formosana*, respectively, were located subcellularly within chloroplasts (Figure 4a) with 19.3% and 9.9% located in cytosol, respectively. In addition, these differentially expressed proteins were further classified according to their metabolic pathways and functions (Figure 4b). The 46 differentially expressed proteins of *C. formosensis* were mainly involved in biological processes such as photosynthesis (67.4%), carbon metabolism (15.2%), amino acid and protein processing (8.7%), signal transduction (4.3%), and stress/defense (4.3%) (Figure 4b). The 15 differentially expressed proteins of *C. obtusa* var. *formosana* were related to photosynthesis (66.7%), carbon metabolism (6.7%), amino acid and protein processing (13.3%), and stress/defense (13.3%) (Figure 4b). The identification results of the differentially expressed proteins of *C. formosensis* and *C. obtusa* var. *formosana* and the tendencies of their protein expressions influenced by light intensity and temperature are listed in Table S2 and Table S3, respectively.

### 2.5. Differentially Expressed Proteins of C. formosensis Sapling at Different Light Intensities

#### 2.5.1. Photosynthesis

**Figure 5:**
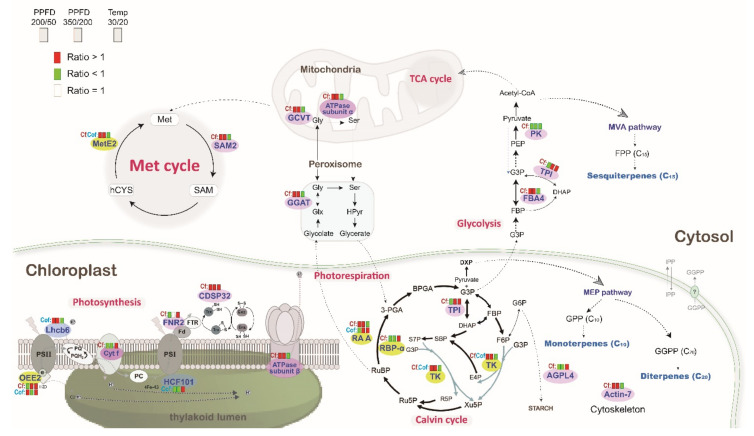
Schematic presentation of different expressed proteins of *C. formosensis* (Cf) and *C. obtusa* var. *formosana* (Cof) under different light intensities and growth temperatures, as shown in Tables S2 and S3. Abbreviations: AGPL4, Glucose-1-phosphate adenylyltransferase large subunit 4; BPGA, 1,3-Bisphosphoglycerate; CDSP32, Thioredoxin-like protein CDSP32; Cyt f, Cytochrome f; DHAP, Dihydroxyacetone phosphate; DXP, 1-Deoxy-D-xylulose 5-phosphate; E4P, Erythrose 4-phosphate; F6P, Fructose 6-phosphate; FBA4, Fructose-bisphosphate aldolase 4; FBP, Fructose 1,6-bisphosphate; Fd, Ferredoxin; FNR2, Ferredoxin-NADP reductase, leaf isozyme 2; FPP, Farnesyl pyrophosphate; FTR, Ferredoxin thioredoxin reductase; G3P, Glyceraldehyde 3-phosphate; G6P, Glucose 6-phosphate; GCVT, Glycine cleavage system T protein; GGAT, Glutamate-glyoxylate aminotransferase; GGPP, Geranylgeranyl pyrophosphate; Glx, Glyoxylate; Gly, Glycine; GPP, Geranyl pyrophosphate; HCF101, Fe-S cluster assembly factor HCF101; hCYS, Homocysteine; Hpyr, Hydroxypyruvate; IPP, Isopentenyl diphosphate; Lhcb6, Chlorophyll a-b binding protein 6A; MEP, methylerythritol 4-phosphate; Met, Methionine; MetE2, 5-Methyltetrahydropteroyltriglutamate-homocysteine methyltransferase 2; MVA, Mevalonate; OEE2, Oxygen-evolving enhancer protein 2; PC, Plastocyanin; PEP, Phosphoenolpyruvate; 3-PGA, 3-Phosphoglycerate; PK, Pyruvate kinase; PPFD, Photosynthetic photon flux density (μmol m^−2^ s^−1^); PQ, Plastoquinone; PQH_2_, Plastoquinol; PSI and PSII, Photosystem I and II; R5P, Ribose 5-phosphate; RA A, Ribulose bisphosphate carboxylase/oxygenase activase A; RBP-α, RuBisCO large subunit-binding protein subunit α; Ru5P, Ribulose 5-phosphate; RuBP, Ribulose 1,5-bisphosphate; S7P, Sedoheptulose 7-phosphate; SAM, S-adenosyl methionine; SAM2, S-adenosyl methionine synthase 2; SBP, Sedoheptulose 1,7-bisphosphate; Ser, Serine; Temp, Temperature (°C); TK, Transketolase; TPI, Triosephosphate isomerase; Trx, Thioredoxins; Xu5P, Xylulose 5-phosphate.

Several photosynthesis-related proteins from *C. formosensis* saplings were found to have different expressions at enhanced light intensities. These proteins included ATP synthase subunit β (ATPase subunit β, spot F325), ferredoxin-NADP reductase, leaf isozyme 2 (FNR2, spot F523), cytochrome f (Cyt f, spot F541), and thioredoxin-like protein CDSP32 (CDSP32, spot F568) (Figure 5 and Table S2). As seen in their order in the pathway of photosynthesis (Figure 5), Cyt f and FNR2 decreased expression at high light intensities. They transport electrons from photosystem II (PSII) to photosystem I (PSI), drive the transformation of energy accumulated in photosynthesis [26], and provide NADPH for the subsequent Calvin cycle reactions [27,28]. CDSP32 and ATPase subunit β increased expression at high light intensity. CDSP32, pertaining to thioredoxin, is normally produced under oxidative stress [29]. ATPase subunit β located on thylakoid membranes is a subunit of ATP synthase. Spot F329, ATPase subunit α in mitochondria, is also a subunit of ATP synthase. ATPase is widely present in chloroplasts and mitochondria in higher plants, and participates in photosynthetic electron transport, as well as oxidative phosphorylation and photophosphorylation reactions. ATPase is a crucial enzyme responsible for energy metabolism and transportation [30]. The aforementioned series of reactions account for the decelerated photosynthetic electron transfer process and photosynthesis in *C. formosensis* saplings at medium or high light intensities through decreased protein expressions of Cyt f and FNR2. Furthermore, they explain how the expression of proteins related to energy metabolism (ATP synthase) is increased to compensate for the lack of energy, and how the harm-removal is accelerated for reactive oxygen species (ROS) (by CDSP32) because of the excessive energy that might be produced by high light intensities. Through a series of energy metabolic regulations, energy was continuously provided for the metabolism process, where *C. formosensis* adapted itself to the increased light intensity.

#### 2.5.2. Calvin Cycle and Carbohydrate Metabolism

The carbon reaction stage of photosynthesis (Calvin cycle) occurs mainly in the stroma of chloroplasts. Carbohydrates synthesized here are required for biosynthetic pathways such as the Shikimate pathway (the biosynthesis of aromatic amino acids and phenylpropanoids) and methylerythritol 4-phosphate (MEP) pathway (the biosynthesis of isoterpenoids) [31,32]. Carbohydrate metabolism was susceptible to environmental changes, and it controlled the metabolism, transformation, and distribution of sugar within plants [33]. In this study, the expression of RuBisCO (ribulose-1,5-bisphosphate carboxylase/oxygenase) large subunit-binding protein subunit α (RBP-α, spots F261-1, F261-2) was influenced by light and temperature (Figure 5 and Table S2). RBP-α participated in the assembly of RuBisCO in the chloroplasts of higher plants. The expression of RBP-α reduced at enhanced light intensity (Table S2), indicating that *C. formosensis* might have rather weak functions of protein folding and protection at high light intensities. In addition, ribulose bisphosphate carboxylase/oxygenase activase A (RA A, spot F437) activated enzymes responsible for catalyzing RuBisCO, which was the most abundant protein in leaves and a key enzyme determining the carbon assimilation rate in photosynthesis [34]. In the carbon fixation reaction, CO_2_ and ribulose 1,5-bisphosphate (RuBP) are first catalyzed to form 3-phosphoglycerate (3-PGA), and the subsequent reactions are further activated [35]. The expression of RA A also affects the carbon assimilation rate and biomass accumulation [28,34]. The expression of RA A protein in *C. formosensis* sapling leaves increased with increasing light intensity (Figure 5 and Table S2), which is consistent with the previous findings [36], suggesting that *C. formosensis* has higher light-use efficiency, which allows saplings to gain more carbon sources and accumulates biomass rapidly.

On the other hand, transketolase (TK, spots F205, F211) is a crucial enzyme in the Calvin cycle. TK differs from other enzymes in its ability to catalyze reversible reactions [37], allowing xylulose 5-phosphate (Xu5P) and ribose 5-phosphate (R5P) to be generated from the process of transferring two carbon molecules by sedoheptulose 7-phosphate (S7P) to glyceraldehyde 3-phosphate (G3P), or enabling Xu5P and erythrose 4-phosphate (E4P) to be yielded from fructose 6-phosphate (F6P). These reactions and TK activity are not only crucial for regeneration and maintenance of RuBP in the Calvin cycle, but also affect the biosynthesis of aromatic amino acids, phenylpropanoids, and isoterpenoids [32,38,39]. In addition, recent research has revealed that TK activity was not regulated by redox, but required the presence of cofactor thiamine pyrophosphate (TPP) [40,41], whereas stress might increase the biosynthesis of TPP and TK activity [32,42]. Thus, under different environmental conditions, the variation in emission rate/released amount of terpenoids might be more susceptible to protein expression. The expression of TK in *C. formosensis* saplings at medium and high light intensities increased significantly (Figure 5 and Table S2), and so did the total emission rate of terpenoids (Figure 3a), which might be due to TK protein regulation.

Pyruvate kinase (PK) and fructose-bisphosphate aldolase 4 (FBA4) are crucial for glycolysis in carbohydrate metabolism. With increase in light intensity, the expressions of PK (spot F314) and FBA4 (spot F438) in *C. formosensis* sapling leaves tended to decrease and increase, respectively (Figure 5 and Table S2). Ruuska et al. (2002) studied *Arabidopsis* seeds and found that PK had isoforms located in both cytosol and plastid, and the two were expressed in opposite directions under the same treatment, meaning that they represented opposite modes of expression in different organelles. The regulation of carbon flux through the metabolic pathway directed from cytosol to plastid was achieved through different expressions of isoforms so as to synthesize substances such as fatty acids and amino acids [43]. FBA4 played a key role in glycolysis, catalyzing fructose 1,6-bisphosphate (FBP) to form dihydroxyacetone phosphate (DHAP) and glyceraldehyde 3-phosphate (G3P) in cytoplasm [44]. At high light intensities, the expressions of FBA4 and PK of cytosol increased and decreased, respectively (Figure 5 and Table S2), which might account for more carbon sources introduced to plastid through reduced expression of cytosol PK in *C. formosensis* saplings. Furthermore, they may maintain normal operation of the Calvin cycle by increasing FBA4. In doing so, the MEP and mevalonate (MVA) biosynthesis pathways can obtain more carbon sources for terpenoid biosynthesis. At high light intensity, emission of terpenoids subsequently increased, contributing to increase in BVOC emission of *C. formosensis* with light intensity.

#### 2.5.3. Other Differentially Expressed Proteins

Four proteins related to amino acid and protein processing showed different expressions. They were glutamate-glyoxylate aminotransferase (GGAT, spots F366, F371) and glycine cleavage system T protein (GCVP, spot F439) for photorespiration, 5-methyltetrahydropteroyltriglutamate-homocysteine methyltransferase 2 (MetE2, spot F172) and S-adenosylmethionine synthase 2 (SAMS2, spots F368, F370-1, F380) for methionine cycle (Met cycle), and actin-7 (spots F370-2, F408) for cytoskeletal protein. The expressions of these proteins all increased significantly with increasing light intensity (Figure 5 and Table S2). GGAT belongs to the photorespiration pathway in peroxisomes. GCVT catalyzed the degradation of the amino acid glycine in mitochondria. Photorespiration involves photosynthesis and metabolism of nitrogen and amino acids as well as protects plants against stress [45,46,47]; Actin-7 was stimulated by external environmental changes, and reinforced to transfer cells and substances to adapt to environmental changes [30,48,49]. Thus, further clarification and examination are required to determine whether increased expression of these proteins at high light intensities in this study act as a protective mechanism for plants against stress at consistent high light intensities in artificially controlled environments.

Overall, in *C. formosensis* saplings at medium and high light intensities, the expressions of photosynthetic proteins Cyt f and FNR2 decreased, whereas the expressions of CDSP32 and ATPase subunit α increased. These changes enhance the energy metabolism, protect plants from oxidative damage, and preserve the energy required for metabolism. In addition, the efficiency of glycolysis increased and more carbon sources tended to distribute in the metabolism of compounds in plastid, which could alleviate the high light intensity-induced energy synthesis and probable damage during carbon reaction. This might account for the increased emission rate of *C. formosensis* saplings at medium and high light intensities. Moreover, increase in terpenoid emission rate might help plants resist high light intensities. With increase in light intensity, the expressions of proteins related to amino acid and protein processing and those of cytoskeletal proteins increased, demonstrating the activation of a series of defense and adaptation mechanisms in *C. formosensis* saplings against changing light intensity.

### 2.6. Differentially Expressed Proteins of C. formosensis Sapling at Different Temperatures

#### 2.6.1. Photosynthesis

When the temperature rose from 20 °C to 30 °C, expression of proteins participating in the light reaction in the photosynthetic system, FNR2 (spot F523), Cyt f (spot F541), CDSP32 (spot F568), and oxygen-evolving enhancer protein 2 (OEE2, spot F647) increased (Figure 5 and Table S2). OEE2, a part of the oxygen-evolving complex (OEC) inside the capsular membrane, was responsible for water photolysis in PSII. Under stress, OEE2 was easily separated from the PSII system, and the increased expression of OEE2 could repair the damage caused by dissociation of these proteins to maintain water photolysis in PSII [50]. In this study, with increase in temperature, more OEE2 of *C. formosensis* saplings expressed, and the photolysis of water also increased, yielding abundant electrons to be fed into PSII. The protein expressions of Cyt f, and those of FNR2 and CDSP32 in the PSI system subsequently increased, thereby enhancing the efficiency of the electron transport chain, and accelerating the transformation of energy accumulated during photosynthesis. However, expression of chaperone proteins ClpC2 (ClpC2, spot F138), ATPase subunit β (spot F325), and ATPase subunit α (spot F329) decreased significantly (Figure 5 and Table S2), indicating that at higher temperature, the efficiency of ATP synthesis in *C. formosensis* saplings was restrained, probably due to enhanced energy transfer that caused ROS accumulation. This situation is similar to the different expressions of proteins in Eucalyptus trees under water stress [51]. The lower BVOC emission rate of *C. formosensis* at 30 °C (Figure 3b) demonstrated possible damage in the photosynthetic system at a higher temperature, thus limiting terpenoid biosynthesis.

#### 2.6.2. Carbon Fixation and Carbohydrate Metabolism

At the increased temperature, the differently expressed proteins related to Calvin cycle reactions included RBP-α (spots F261-1, F261-2), RA A (spot F437), CBBY-like protein (Cbby, spot F607-2), and triosephosphate isomerase (TPI, spot F607-3) (Figure 5). The expression of these proteins all increased significantly with increase in temperature except for that of RA A (Table S2). The decrease in RA A expression indicated damaged redox balance in *C. formosensis* sapling leaves at higher temperature and restrained photosynthesis, whereas increased expression of chaperone protein RBP-α prevented and reversed inappropriate protein interaction, facilitating correct protein folding. Cbby is responsible for converting XuBP (xylulose 1,5-bisphosphate), a potent inhibitor of RuBisCO, to the non-inhibitory Xu5P [52]. The elevated Cbby expression was likely involved in maintaining RuBisCO’s proper function caused by higher temperature. In glycolysis, TPI (triosephosphate isomerase) catalyzed the transformation between DHAP and G3P, and it was indispensable for effective generation of energy [53]. Glycolysis plays a crucial role in the dynamic regulation of the carbohydrate metabolic pathway, and the maintenance of balanced mid-products not only avoided toxic damage caused by excessive accumulation but also preserved the stable glycolysis metabolism cycle. The regulation of enzymes is thus extremely crucial in terms of maintaining a balanced metabolism in plants during cell growth or environmental changes [53,54]. As for other proteins related to carbohydrate metabolism, the expressions of TK, inositol-3-phosphate synthase (MIP synthase, spot F304), PK, and FBA4 all decreased significantly at higher temperature (Figure 5 and Table S2). These proteins are all related to the glycolysis metabolic pathway and further extended to terpenoid biosynthesis. Thus, the terpenoid emission rate of *C. formosensis* saplings decreased significantly at higher temperature, probably due to restrained terpenoid biosynthesis.

### 2.7. Differentially Expressed Proteins of C. obtusa var. formosana Saplings at Different Light Intensities and Temperatures

In contrast to *C. formosensis* saplings, in which a series of protein reactions were activated to respond to environmental changes, *C. obtusa* var. *formosana* saplings had fewer differentially expressed proteins regardless of whether at increased light intensity or temperature conditions (Figure 5 and Table S3). Furthermore, the fold changes of proteins in *C. obtusa* var. *formosana* were quite often smaller than those in *C. formosensis*. The expressions of MetE2 (spot O79), TK (spot O98), and chlorophyll a-b binding protein 6A (Lhcb6, spot O390) increased with increasing light intensity. Although the expression of RA A (spot O347) was a little bit lower in medium light intensity (PPFD = 200 μmol m^−2^ s^−1^), its expression also increased in high light intensity (PPFD = 350 μmol m^−2^ s^−1^). In contrast, the expression of Fe-S cluster assembly factor HCF101 (HCF101, spot O162) decreased with increasing light intensity (Figure 5 and Table S3). Among these proteins, Lhcb6 was one of the light-harvesting chlorophyll a/b-binding (LHC) proteins, which not only serves as a photon receiver and transmitter, but also has the function of photoprotection [55,56]. Recent research revealed that three minor chlorophyll-binding antenna proteins, namely Lhcb4, Lhcb5, and Lhcb6, exerted critical regulation and protection against oxidative damage produced in plants under natural light [56]. Their findings indicated that the oxygen-release capacities of mutants lacking Lhcb4 and Lhcb6 were slightly decreased, and the photosynthetic rates and adaptabilities of mutants lacking Lhcb5 or Lhcb6 were also affected. The mutants lacking Lhcb6 had the lowest antioxidant enzyme activities, and with the accumulation of abundant superoxide radicals, they suffered more serious oxidative damage compared with other mutants. Thus, Lhcb6 is a crucial protein providing photoprotection to the plant body and alleviating oxidative stress [56]. At increased light intensity, Lhcb6 might first exert the function of defense in photosystem, avoiding the probable oxidative stress suffered by *C. obtusa* var. *formosana* saplings at high light intensities. On the other hand, HCF101, an indispensable member participating in PSI, decreased its expression at high light intensities. HCF101 is responsible for assembling iron-sulfur (4Fe-4S) clusters and transferring them to PSI and ferredoxin-thioredoxin reductase (FTR) [50]. At increased light intensity, the expression of HCF10 decreased (Figure 5 and Table S3), indicating that at high light intensities with a consistent manually controlled environment, the completeness of chloroplasts and the photosynthetic rate of plants might be restrained. The expression of TK (spot O98) and RA A (spot O347) of *C. obtusa* var. *formosana* at high light intensities is consistent with that of *C. formosensis*, indicating that the increased BVOC emission rate of *C. obtusa* var. *formosana* saplings in high light intensity might be the results from the upregulated TK and RA A proteins. This could shuffle the carbon sources of the plant body toward the terpenoid biosynthesis.

Photosystem proteins, HCF101 (spot O162) and OEE2 (spot O397), signal transduction protein, guanine nucleotide-binding protein subunit β-like protein (GB1, spot O318), and stress defense-related protein, probable L-ascorbate peroxidase 5 (APX5, spot O328), were all increased at higher temperature (Figure 5 and Table S3). OEE2 (spot O397), protein responsible for the water photolysis in the photosynthetic system PSII, and HCF101, factor responsible for the Fe-S cluster assembly, were both increased at higher temperature, indicating that these two proteins participated in improving or facilitating the maintenance of photosynthesis efficiency of plants at higher temperature and exerting the function of thermotolerance. These results are consistent with the simultaneous enhancement of HCF101 and OEE2 expressed in *Parthenium hysterophorus* under drought and salt stress [50]. In addition, the decreased expressions of Lhcb6 (spot O390), TK (spot O98), and MetE2 (spot O79) proteins (Figure 5 and Table S3) revealed that higher temperature might restrain the photosynthetic rate and carbon metabolism. Thus, the carbon sources provided for the MEP biosynthetic pathway decreased significantly, which might be a reason for the decreased release of BVOCs from *C. obtusa* var. *formosana* saplings at higher temperature (Figure 3b).

## 3. Materials and Methods

### 3.1. Plant Materials

Three-year-old saplings of *Chamaecyparis formosensis* and *C. obtusa* var. *formosana* obtained from the nursery at Chu-Yun Mountain, Dongshih Forest District Office, Taichung, Taiwan (24°14′03.9″ N 120°54′14.5″ E, elevation: 1000 m) were used in this study. Saplings were planted in pots filled with a mixture of perlite, vermiculite, and peat moss (1:1:1 *v*/*v*/*v*). Simple sequence repeat (SSR) genotyping (Supplementary Methods and Table S4) was employed to test the genetic diversity of 56 saplings of *C. formosensis* and 55 saplings of *C. obtusa* var. *formosana*. Saplings with similar genetic backgrounds were selected (Figure S3), and their BVOC chemotypes were further analyzed with static-headspace (static-HS) extraction (Supplementary methods and Figure S4). Nine saplings with similar genetic backgrounds and BVOCs were finally selected and grown in a phytotron-controlled environment (80% RH, 12 h light/12 h dark cycle) under each different condition (temperature: 20 °C or 30 °C; light intensities or photosynthetic photon flux densities (PPFDs): 50, 200, or 350 μmol m^−2^ s^−1^) for two weeks. The conditions chosen were based on previous publications [5,20] considering the temperature and light intensity ranges in the natural habits of these cypresses. When the 9 saplings were exposed to individual light intensity treatment, the temperature was 20 °C; when the individual temperature treatment at 20 °C or 30 °C was performed, these 9 saplings were exposed under 200 μmol m^−2^ s^−1^ light intensity. After that, their CO_2_ assimilation rates and BVOC emissions were measured. Proteomic analyses were further conducted on three out of the nine healthy saplings.

### 3.2. Measurement of the Net CO_2_ Assimilation Rate

The net CO_2_ assimilation rates of nine saplings of *C. formosensis* and *C. obtusa* var. *formosana* were measured using a portable photosynthesis system (LI-6400, LI-COR, Lincoln, NE, USA) equipped with a standard 2 × 3 cm^2^ leaf chamber, a CO_2_ injector, and a red/blue LED light source (6400-02B). Leaves were photographed before photosynthetic measurements and their leaf areas were determined using ImageJ NIH image processing software (Image J, NIH, Bethesda, MD, USA) [57]. The CO_2_ concentration within the cuvette was controlled at 400 ppm, and the flow rate was set at 500 μmol s^−1^.

### 3.3. BVOC Emission of Saplings in Controlled Environment

BVOC sampling procedure and quantitative method were as described in Chen et al. (2019) [21]. The healthy leaves were carefully enclosed in a glass sampling chamber (Figure 2). The BVOCs emitted from leaves to the chamber were collected with sampling tubes (200 mg Tenax TA, mesh 60/80, Supelco, Bellofonte, PA, USA), installed in a modified autosampler (STS-25^®^; PerkinElmer, Waltham, MA, USA) with 150 mL min^−1^ flow rate for BVOCs to flow into the absorbent. Each sampling tube collected BVOCs emitted for a period of 60 min, every h during daytime (04:00–16:00) and every 2 h during nighttime (18:00–04:00), with 8.25 L BVOCs collected per sample tube. BVOC sampling tubes were brought back to laboratory every day for analysis on contents and concentrations of BVOCs. The sample tube was analyzed using gas chromatography mass spectrometer (GC-MS) (Clarus 600 GC-MS system, PerkinElmer Instruments, USA) equipped with a thermal desorption system (Turbo Matrix ATD, PerkinElmer Instruments, USA). The data of 17 sampling tubes in one day were combined to represent the BVOCs emitted by the sapling. After sampling, shoots in the sampling chamber were cut, then placed in 100 °C for 48 h, and finally weighed to obtain the absolute dry biomass.

### 3.4. BVOC Qualitative Analysis

Terpenoids were separated using a fused silica capillary column (DB-5 ms, length 30 m, i.d. 0.25 mm, film 0.25 μm). The injection temperature was 250 °C. The flow rate of carrier gas, He, was 1 mL min^−1^. The MS detector was set up at 230 °C in scan mode with *m*/*z* ranging from 40 to 350 a.m.u. Initial oven temperature was increased from 50 °C to 90 °C at 5 °C min^−1^, to 160 °C at 3 °C min^−1^, then ramped to 260 °C at 35 °C min^−1^, and maintained at 260 °C for 5 min. Identification of terpenoids was made by comparison with mass spectra of NIST MS Search (National Institute of Standards and Technology, version 2.0) and Wiley/NBS Registry of Mass Spectral Database (Version 7.0). The arithmetic index (AI) with mass spectra library and the reference AI (rAI) were also used in identification of terpenoids [58], and the AI was calculated using the formula:

AI = 100 × [n + (RT_(x)_ − RT_(n)_)/(RT_(n + 1)_ − RT_(n)_)] (1)

RT_(n)_ and RT_(n + 1)_: the retention time of *n*-Alkanes and (*n* + 1)-Alkanes

RT_(x)_: the retention time of unknown compound, RT_(n)_ ≤ RT_(x)_ ≤ RT_(n + 1)_

### 3.5. BVOC Quantitative Analysis

The spectra of external standards for commercially available compounds were employed to quantify the absolute content of BVOCs, including α-pinene (98%, Acros), β-pinene (98%, Acros), δ-3-carene (90%, Acros), α-terpinene (90%, Acros), limonene (92%, Acros), terpinen-4-ol (97%, Acros), α-phellandrene (90%, TCI), terpinolene (95%, TCI), sabinene (90%, ChromaDex), *trans*-β-farnesene (90%, ChromaDex), α-cedrene (99%, Fluka), thujopsene (97%, Fluka), camphene (96%, ICN), β-myrcene (90%, Sigma), *trans*-β-ocimene (90%, SAFC), and γ-terpinene (98%, Aldrich). Compounds α-phellandrene and *trans*-β-farnesene were used for quantifying β-phellandrene and (*E*,*E*)-α-farnesene whose standards were not available. Kaur-16-ene was collected and purified from the extract of *Cryptomeria japonica* leaves using HPLC in the laboratory. Kaur-16-ene was used for quantifying diterpenoids without standard. In addition, an internal standard (Chlorobenzene d5) was added into all samples to compensate the systematic error and to obtain good quantitative results.

### 3.6. Plant Protein Extraction and 2D-Difference Gel Electrophoresis (2D-DIGE)

Partial leaves used in BVOC sampling were cut and preserved in liquid nitrogen, and their proteins were then extracted with TCA (trichloroacetic acid)/acetone [14,15]. The isolated protein samples were grouped according to Table S1, and labeled with DIGE-Kit (Visual Protein, Taipei, Taiwan). Thirty-microgram protein samples were used for individual sample labeling using Cy3 or Cy5, and the internal standard was an equal amount mixture of all samples used in the experiment and labeled with Cy2. The labeled samples were then rehydrate-loaded onto an IPG Strip (13 cm, 3–10 NL) (GE Healthcare, Chicago, USA) at 25 °C for 10 h. An Ettan IPGphor system (GE Healthcare, Chicago, IL, USA) was employed for isoelectric focusing (IEF) using the program: 500 V (500 Vh), 1 kV (800 Vh, gradient), 8 kV (11300 Vh, gradient), and finally 8 kV up to 18,000 Vhs. Then, the strips were equilibrated in equilibration buffer I (50 mM Tris–HCl (pH 8.8), 6 M urea, 30% (*v*/*v*) glycerol, 2% (*w*/*v*) SDS, 2% DTT) for 15 min, and subsequently in equilibration buffer II (50 mM Tris–HCl (pH 8.8), 6 M urea, 30% (*v*/*v*) glycerol, 2% (*w*/*v*) SDS, 2.5% iodoacetamide) for 15 min with gentle shaking. After equilibration, the strips were transferred to a 12.5% (*w*/*v*) SDS-PAGE-containing protein ladder (10 kDa–170 kDa). The IEF strip was sealed on gels with 0.5% (*w*/*v*) agarose containing bromophenol blue as the tracking dye, and electrophoresis was performed with an Hoefer SE 600 Ruby System (GE Healthcare, Chicago, IL, USA) until the bromophenol blue moved to the gel bottom. After electrophoresis, images were visualized using Typhoon Trio scanner (GE Healthcare, Chicago, IL, USA), with the excitation laser/the emission filter wavelength of each dye: Cy2 (Blue, 488/520 nm), Cy3 (Green, 532/580 nm), Cy5 (Red, 633/670 nm). The images were imported into Progenesis SameSpots™ 2D software (Version 4.1, Nonlinear Dynamics, Newcastle, UK) for analysis. Spots with volumes > or < 1.00-fold changes in their relative abundance with a *p*-value less than 0.05 in *t*-test between different treatment groups were considered as differentially expressed proteins.

### 3.7. LC-MS/MS and Protein Identification

Selected spots excised from the gels were in-gel digested, and sent for analysis using an Orbitrap Fusion Lumos mass spectrometer (Thermo Fisher Scientific, Bremen, Germany) at Mass Spectrometry Platform, NTU Instrumentation Center, National Taiwan University. Identification of the selected protein spots and database search were performed as described by Chen et al. (2015) [59]. In brief, MASCOT software (www.matrixscience.com (accessed on 30 April 2022)) was used for protein identification and comparison with the National Center for Biotechnology Information (NCBI) nonredundant protein sequence database. The identified proteins whose scores exceeded 50 and *p* < 0.05 were considered significant hits. Gene ontology annotation of these proteins was performed using the Blast2GO program (Version 5.2.5).

### 3.8. Statistical Analysis

The differences in net CO_2_ assimilation rate among the three light intensities or two temperatures were examined using one-way repeated measurement ANOVA. If the differences among the three light intensities were significant (*p* < 0.05), paired *t*-test was conducted as the post hoc test. BVOC emission among treatments were analyzed using one-way ANOVA, and differences with *p* < 0.05 were considered significant. Multiple comparison was performed by the Scheffe’s test. These statistical analyses were conducted using the SPSS program package (Statistical Product and Service Solutions, Version 17.0).

## 4. Conclusions

Under a controlled growing environment, the net CO_2_ assimilation rates and BVOC emission rates of both *C. formosensis* and *C. obtusa* var. *formosana* saplings increased with the increases of light intensity. The magnitude of change was higher in *C. formosensis*. However, *C. obtusa* var. *formosana* had considerably increased sesquiterpenoid and diterpenoid emission in BVOC under high light intensity than *C. formosensis*. Both species grown at 30 °C had significantly lower BVOC emission rates than those grown at 20 °C. Proteomic analyses reveal that compared to *C. formosensis* saplings, *C. obtusa* var. *formosana* saplings had fewer differentially expressed proteins in terms of protein species and fold changes in response to the environmental changes. Both species showed significant changes of the carbon fixation-related proteins, thymidine kinase (TK) and ribulose bisphosphate carboxylase/oxygenase activase A (RA A), and the photosynthesis-related oxygen-evolving enhancer protein-2 (OEE2). These might be the regulatory factors that affect BVOCs’ emission of both species under different environments. Further in-depth examinations of these regulatory mechanisms should be performed to determine whether these changes/adaptive mechanisms affect the saplings’ subsequent survival and distribution.

## Data Availability

The data that support the findings of this study are available in the main text and the Supplementary Materials.

## Supplementary Materials

The following are available online at https://www.mdpi.com/article/10.3390/plants11121535/s1, Supplementary methods, Figure S1: Venn diagram showing the number and relationship of proteins differentially expressed for the light intensity and temperature comparisons from the *C. formosensis*. PPFD, photosynthetic photon flux density or light intensity (μmol m^−2^ s^−1^), Figure S2: Venn diagram showing the number and relationship of proteins differentially expressed for the light intensity and temperature comparisons from the *C. obtusa* var. *formosana*. PPFD, photosynthetic photon flux density or light intensity (μmol m^−2^ s^−1^), Figure S3: Genetic structure of populations of (a) *C. formosensis*, and (b) *C. obtusa* var. *formosana* saplings, Figure S4: Cluster analysis of the constituents of volatile compounds from (a) *C. formosensis*, and (b) *C. obtusa* var. *formosana* saplings using static-headspace, Table S1: Schematic representation of 2D-DIGE experimental design, Table S2: Identification results of different expressed proteins of sapling leaves of *C. formosensis* under different light intensities and temperatures, Table S3: Identification results of different expressed proteins of sapling leaves of *C. obtusa* var. *formosana* under different light intensities and temperatures, Table S4: List of primer sequences used in this study.
