# Supplementary material for: Biogenic Volatile Organic Compounds and Protein Expressions of Chamaecyparis formosensis and Chamaecyparis obtusa var. formosana Leaves under Different Light Intensities and Temperatures
Source: Plants (Basel). 2022 Jun 8;11(12):1535. doi: 10.3390/plants11121535 (PMC9231097; doi:10.3390/plants11121535)
Supplement: Supplementary file 1 [file plants-11-01535-s001.zip › plants-1726955-supplementary.pdf]

Supplementary Materials

Biogenic volatile organic compounds and protein expressions of *Chamaecyparis formosensis* and *Chamaecyparis obtusa* var. *formosana* leaves under different light intensities and temperatures

Ying-Ju Chen[1,2], Ya-Lun Huang[1,†], Yu-Han Chen[1], Shang-Tzen Chang[1*], Ting-Feng Yeh[1*]

[1] School of Forestry and Resource Conservation, National Taiwan University, Taipei 10617, Taiwan

[2] Division of Forest Chemistry, Taiwan Forestry Research Institute, Taipei 10070, Taiwan

[†] Current address: Department of Life Science, National Taiwan University, Taipei 10617, Taiwan

[*]Correspondence: peter@ntu.edu.tw (S.-T.C.); stfyeh@ntu.edu.tw (T.-F.Y.).

Supplementary Material Content

Supplementary methods..Page S2 - S4
Supplementary Tables...Page S5 - S12
Supplementary Figures ....Page S13 - S17

Supplementary methods

Simple sequence repeat (SSR) genotyping

In order to reduce the effects of individual variance, different saplings from the same species with similar genetic structures were selected by using SSR genotyping technique. The molecular marker used in this study was short tandem repeat sequence. All molecular markers were provided by Dr. Kuo-Fang Chung, Biodiversity Research Center, Academia Sinica, Taiwan. In each species, different sets of molecular markers were used. *C. formosensis*: No4 (159 bp), No35 (193 bp), No47 (153 bp), No88 (130 bp), and *C. obtusa* var. *formosana*: No76 (156 bp), No77 (170 bp), No99 (177 bp). The primer sequences were shown in Table S4 [1]. Genomic DNA of both tree species were isolated and amplified by polymerase chain reaction (PCR) using the aforementioned primers which were fluorescent-labelled with either 6-carboxyfluorescein (6-FAM) or 4,5-dichloro-dimethoxy-fluorescein (JOE). The genotyping of PCR product was analyzed with the automatic sequencer with fluorescent capillary (3730XL DNA Analyzer, Genomics, New Taipei City, Taiwan), and then was interpreted by GeneMapper 4.0 (Applied Biosystems, Thermo Fisher Scientific, USA).

The genetic structure and composition of 56 *C. formosensis* and 55 *C. obtusa* var. *formosana* saplings were assigned by the genotyping results using the Structure Software (Version 2.3.4, Pritchard Lab, Stanford University, USA) [2]. The optimal number of clusters (K) and adjust ΔK values were calculated according to Evanno method [3] and the network software Structure Harvester Version 0.6.8 [4].

According to the results of Bayesian clustering analysis, the optimal numbers of clusters (K) of *C. formosensis* and *C. obtusa* var. *formosana* were 13 and 10, respectively. The genetic backgrounds of different saplings for both species were presented with different color combinations (Figure S3), and saplings with similar color combinations indicated that they tended to share similar genetic backgrounds [5]. According to the results in Figure S3, 36 *C. formosensis* and 47 *C. obtusa* var. *formosana* saplings were further selected for subsequent BVOC chemotype analysis.

BVOC chemotype analysis

To further differentiate the chemotypes of the 36 *C. formosensis* and 47 *C. obtusa* var. *formosana*, the volatile terpenoid compounds of leaves from these saplings were analyzed according the static-headspace (Static-HS) method described by Chen et al. (2015) [6]. Twenty mg of leaves from individual sapling was cut and put in a 22 mL sample vial. The BVOCs of these samples were extracted with Turbomatrix 40 Static-HS (PerkinElmer Inc., USA) and analyzed with gas chromatography-mass spectrometry (Clarus 600 GC-MS system, PerkinElmer Instruments, USA). Headspace equilibrium temperature was set to 150°C for 30 min. The released volatile components by heating were directly introduced into the GC-MS for analysis. The temperature of injection needle and the transfer line were set 160°C and 170°C, respectively, and the injection time was 0.02 min.

Cluster analysis was used to analyze the chemical component of BVOCs collected by Static-HS from *C. formosensis* and *C. obtusa* var. *formosana* saplings. The similarity was evaluated by Euclidean distance with UPGMA (Unweighted Pair Group Method with Arthmetic Averages) by MVSP (Multi-Variate Group Statistical Package). Smaller Eulidean distance between two samples means more similar BVOCs components. The results of cluster analysis (Figure S4) indicated that the similarities of chemical composition among the *C. formosensis* all exceeded 95%. On the other hand, although the diversity of chemical composition among individual *C. obtusa* var. *formosana* was greater, the similarities among them were still higher than 60%. To select individual saplings with more homogeneous chemical compositions for the subsequent tests to reduce the probable interference of treatment effects caused by individual variation, nine *C. formosensis* plants (among which the similarity exceeded 97%) and nine *C. obtusa* var. *formosana* plants (among which the similarity exceeded 76%) were selected for subsequent physiological and proteomic experiments.

References for supplementary methods

1. Huang, C.J.; Chu, F.H.; Liu, S.C.; Tseng, Y.H.; Huang, Y.S.; Ma, L.T.; Wang, C.T.; You, Y.T.; Hsu, S.Y.; Hsieh, H.C.; Chen, C.T.; Chao, C.H. Isolation and characterization of SSR and EST-SSR loci in *Chamaecyparis formosensis* (Cupressaceae). *Appl. Plant Sci.* **2018**, *6*, e01175, doi:10.1002/aps3.1175.
2. Pritchard, J.K.; Stephens, M.; Donnelly, P. Inference of population structure

using multilocus genotype data. *Genetics* **2000**, *155*, 945-959, doi:10.1093/genetics/155.2.945.

3. Evanno, G.; Regnaut, S.; Goudet, J. Detecting the number of clusters of individuals using the software STRUCTURE: a simulation study. *Mol. Ecol.* **2005**, *14*, 2611-2620, doi:10.1111/j.1365-294X.2005.02553.x.
4. Earl, D.A.; Vonholdt, B.M. STRUCTURE HARVESTER: a website and program for visualizing STRUCTURE output and implementing the Evanno method. *Conserv. Genet. Resour.* **2012**, *4*, 359-361, doi:10.1007/s12686-011-9548-7.
5. Hsieh, H.L.; Ma, L.T.; Wang, S.Y.; Chu, F.H. Cloning and expression of a sesquiterpene synthase gene from *Taiwania cryptomerioides*. *Holzforschung* **2015**, *69*, 1041-1048, doi:10.1515/hf-2014-0279.
6. Chen, Y.J.; Lin, C.Y.; Cheng, S.S.; Chang, S.T. Rapid discrimination and feature extraction of three *Chamaecyparis* species by static-HS/GC-MS. *J. Agric. Food Chem.* **2015**, *63*, 810-820, doi:10.1021/jf505587w.

Supplementary Tables

Table S1. Schematic representation of 2D-DIGE experimental design

Table S2. Identification results of different expressed proteins of sapling leaves of *C. formosensis* under different light intensities and temperatures

Table S3. Identification results of different expressed proteins of sapling leaves of *C. obtusa* var. *formosana* under different light intensities and temperatures

Table S4. List of primer sequences used in this study

Table S1. Schematic representation of 2D-DIGE experimental design

Gel	Cy2	Cy3[a]	Cy5[a]
1	Internal standard	20°C M-1	30°C M-1
2	Internal standard	20°C M-2	20°C L-1
3	Internal standard	20°C H-1	20°C M-3
4	Internal standard	30°C M-2	20°C L-2
5	Internal standard	30°C M-3	20°C H-2
6	Internal standard	20°C L-3	20°C H-3

[a] L: PPFD (Photosynthetic photon flux density or light intensity) = 50 µmol m^{-2} s^{-1}; M: PPFD = 200 µmol m^{-2} s^{-1}; H: PPFD = 350 µmol m^{-2} s^{-1}.

Table S2. Identification results of different expressed proteins of sapling leaves of *C. formosensis* under different light intensities and temperatures

Functional annotation/Biological process	Sample ID	Short name	Protein name	Accession No.	Fold changes[a]		
					PPFD 200/50	PPFD 350/200	Temp 30/20
Photosynthesis							
Photosynthesis	F138	ClpC2	Chaperone protein ClpC2	Q9SXJ7	0.97	1.09	0.79
Photosynthesis	F325	ATPase subunit β	ATP synthase subunit β	Q9BA85	1.42	1.01	0.51
Photosynthesis	F523	FNR2	Ferredoxin-NADP reductase, leaf isozyme 2	Q6ZFJ3	0.61	1.00	1.67
Photosynthesis	F541	Cyt f	Cytochrome f	B1VKB7	0.29	0.86	3.96
Photosynthesis	F568	CDSP32	Thioredoxin-like protein CDSP32	Q9SGS4	1.31	1.27	1.17
Photosynthesis	F647	OEE2	Oxygen-evolving enhancer protein 2	P85189	0.75	1.16	1.40
Calvin cycle	F261-1	RBP-α	RuBisCO large subunit-binding protein subunit α	P08926	0.62	0.83	1.68
Calvin cycle	F261-2	RBP-α	RuBisCO large subunit-binding protein subunit α	P08926	0.62	0.83	1.68
Calvin cycle	F345	RBCL	Ribulose bisphosphate carboxylase large chain	P48696	0.93	0.99	0.74
Calvin cycle	F354	RBCL	Ribulose bisphosphate carboxylase large chain	P48696	0.92	0.97	0.63
Calvin cycle	F437	RA A	Ribulose bisphosphate carboxylase/oxygenase activase A	Q40073	1.38	1.20	0.81
Calvin cycle	F489	RBCL	Ribulose bisphosphate carboxylase large chain	A6MMV2	0.42	1.01	2.57
Calvin cycle	F518	RBCL	Ribulose bisphosphate carboxylase large chain	Q32026	0.44	0.92	2.19
Calvin cycle	F521	RBCL	Ribulose bisphosphate carboxylase large chain	Q3V526	1.35	1.06	0.62
Calvin cycle	F534	RBCL	Ribulose bisphosphate carboxylase large chain	P48696	0.26	0.63	3.14
Calvin cycle	F539	RBCL	Ribulose bisphosphate carboxylase large chain	Q32026	0.27	0.85	3.65
Calvin cycle	F561	RBCL	Ribulose bisphosphate carboxylase large chain	P31184	1.07	1.17	0.73

Table S2. Identification results of different expressed proteins of sapling leaves of *C. formosensis* under different light intensities and temperatures (*continued*)

**Functional annotation/Biological process**	**Sample ID**	**Short name**	**Protein name**	**Accession No.**	**Fold change**[a]		
					**PPFD 200/50**	**PPFD 350/200**	**Temp 30/20**
Calvin cycle	F575	RBCL	Ribulose bisphosphate carboxylase large chain	A8W3D6	0.65	1.20	1.63
Calvin cycle	F599	RBCL	Ribulose bisphosphate carboxylase large chain	P24671	1.25	1.10	0.70
Calvin cycle	F607-1	RBCL	Ribulose bisphosphate carboxylase large chain	P34915	0.76	1.06	1.43
Calvin cycle	F607-2	Cbby	CBBY-like protein	Q94K71	0.76	1.06	1.43
Calvin cycle	F607-3	TPI	Triosephosphate isomerase	P48496	0.76	1.06	1.43
Calvin cycle	F613	RBCL	Ribulose bisphosphate carboxylase large chain	P48696	0.73	0.90	0.99
Calvin cycle	F626	RBCL	Ribulose bisphosphate carboxylase large chain	P48695	0.35	1.25	4.06
Calvin cycle	F682	RBCL	Ribulose bisphosphate carboxylase large chain	P36479	1.24	1.04	0.76
Calvin cycle	F720	RBCL	Ribulose bisphosphate carboxylase large chain	P48696	2.34	0.88	1.77
Calvin cycle	F728	RBCL	Ribulose bisphosphate carboxylase large chain	P48696	2.92	0.90	1.65
Photorespiration	F329	ATPase subunit α	ATP synthase subunit α	P0C522	1.32	1.06	0.66
Photorespiration	F366	GGAT	Glutamate-glyoxylate aminotransferase	Q9S7E9	1.25	1.10	0.65
Photorespiration	F371	GGAT	Glutamate-glyoxylate aminotransferase 2	Q9S7E9	1.38	1.10	0.69
Photorespiration	F439	GCVT	Glycine cleavage system T protein	P54260	1.23	1.18	0.77
Carbon metabolism							
Calvin cycle	F205	TK	Transketolase	Q7SIC9	1.22	1.28	0.70
Calvin cycle	F211	TK	Transketolase	Q43848	1.38	1.09	0.55
Glycolysis	F304	MIP synthase	Inositol-3-phosphate synthase	Q9FYV1	1.33	1.19	0.70

Table S2. Identification results of different expressed proteins of sapling leaves of *C. formosensis* under different light intensities and temperatures (*continued*)

Functional annotation/Biological process	Sample ID	Short name	Protein name	Accession No.	Fold change[a]		
					PPFD 200/50	PPFD 350/200	Temp 30/20
Glycolysis	F314	PK	Pyruvate kinase	Q42954	0.78	0.78	0.80
Glycolysis	F393	AGPL4	Glucose-1-phosphate adenylyltransferase large subunit 4	Q0D7I3	0.40	0.95	2.48
Glycolysis	F394	AGPL4	Glucose-1-phosphate adenylyltransferase large subunit 4	Q0D7I3	0.63	0.90	2.12
Glycolysis	F438	FBA4	Fructose-bisphosphate aldolase 4	F4KGQ0	1.13	1.14	0.82
Amino acid and protein processing							
Met cycle	F172	MetE2	5-Methyltetrahydropteroyltriglutamate-homocysteine methyltransferase 2	Q2QLY4	1.92	1.19	0.47
Met cycle	F368	SAMS2	S-Adenosylmethionine synthase 2	Q9FUZ1	1.57	1.14	0.49
Met cycle	F370-1	SAMS2	S-Adenosylmethionine synthase 2	Q9FVG7	1.30	1.12	0.74
Met cycle	F380	SAMS2	S-Adenosylmethionine synthase 2	A9NYY0	1.76	1.23	0.53
Signal transduction							
Cytoskeleton	F370-2	Actin-7	Actin-7	P53492	1.30	1.12	0.74
Cytoskeleton	F408	Actin-7	Actin-7	P53492	1.21	1.20	0.82
Stress/defense							
Stress/defense	F504	-	Probable aldo-keto reductase 1	B8ASB2	0.66	1.12	1.59
Stress/defense	F520	PLP synthase subunit PDX1	Probable pyridoxal 5'-phosphate synthase subunit PDX1	Q39963	0.77	1.06	1.64

[a] PPFD: light intensity, µmol m^{-2} s^{-1}; Temp: temperature, °**C**.

Table S3. Identification results of different expressed proteins of sapling leaves of *C. obtusa* var. *formosana* under different light intensities and temperatures

Functional annotation/Biological process	Sample ID	Short name	Protein name	Accession No.	Fold change[a]		
					PPFD 200/50	PPFD 350/200	Temp 30/20
Photosynthesis							
Photosynthesis	O162	HCF101	Fe-S cluster assembly factor HCF101	Q6STH5	0.89	0.95	1.21
Photosynthesis	O390	Lhcb6	Chlorophyll a-b binding protein 6A	P12360	1.35	1.02	0.94
Photosynthesis	O397	OEE2	Oxygen-evolving enhancer protein 2	P85189	1.15	0.79	1.04
Calvin cycle	O133	RBP-β	RuBisCO large subunit-binding protein subunit β	P08927	0.99	1.12	0.98
Calvin cycle	O347	RA A	Ribulose bisphosphate carboxylase/oxygenase activase A	Q40073	0.91	1.13	1.21
Calvin cycle	O351-1	RBCL	Ribulose bisphosphate carboxylase large chain	P48696	0.77	1.04	1.02
Calvin cycle	O351-2	RBCL	Ribulose bisphosphate carboxylase large chain	Q3V526	0.77	1.04	1.02
Calvin cycle	O353	RBCL	Ribulose bisphosphate carboxylase large chain	A0ZZ43	0.74	0.95	1.23
Calvin cycle	O402	RBCL	Ribulose bisphosphate carboxylase large chain	P24680	1.29	1.02	0.63
Calvin cycle	O405	RBCL	Ribulose bisphosphate carboxylase large chain	Q37328	0.65	1.01	1.31
Carbon metabolism							
Calvin cycle	O98	TK	Transketolase, chloroplastic	Q43848	1.03	1.17	0.92
Amino acid and protein processing							
Met cycle	O79	MetE2	5-methyltetrahydropteroyltriglutamate-homocysteine methyltransferase	Q42662	1.27	1.23	0.71

Table S3. Identification results of different expressed proteins of sapling leaves of *C. obtusa* var. *formosana* under different light intensities and temperatures (*continued*)

**Functional annotation/Biological process**	**Sample ID**	**Short name**	**Protein name**	**Accession No.**	**Fold change[a]**		
					**PPFD 200/50**	**PPFD 350/200**	**Temp 30/20**
Signal transduction							
Cytoskeleton	O318	GB1	Guanine nucleotide-binding protein subunit β-like protein	O24076	0.65	1.18	1.37
Stress/defense							
Stress/defense	O328	APX5	Probable L-ascorbate peroxidase 5	P0C0L0	1.75	0.67	1.15
Stress/defense	O343	40S RP S3-1	40S ribosomal protein S3-1	Q9SIP7	1.54	0.87	0.87

[a] PPFD: light intensity, μmol m^{-2} s^{-1}; Temp: temperature, **°C**.

Table S4. List of primer sequences used in this study

Primer No.	Primer Name	Forward primer sequence (5' - 3')	Reverse primer sequence (5' - 3')	Predicted length (bp)	Fluorescent Dye[a]
4	AGAT78_137	ACGCTTTCACCTCCATAGTA	AATAGGGGTTTCCCTACATC	159	6-FAM
35	GATA13_3	GGAGAAAGGAGTGTCACAAG	AACTCATTCCTTCTCCCTCT	193	6-FAM
47	TATC78_25	CCTCTCTCTCACCCCTCTAT	TCTGTATGAGTGTTGCTCCA	153	JOE
76	TATC27_29	TCTCATTCAAGTGGTATGTT	TCATCTTCACGAACCAAGA	156	JOE
77	TATC74_82	TGACGTGTCAATCTTTTGG	AAGAAAAGGTTGCAATGGT	170	6-FAM
88	TATC97_18	GCTTCATCGTCCCTAAGTT	TTCTGTTCTTGCAAATTGTT	130	6-FAM
99	TATG74_148	GGGAGCTGTAGGGAGATGAA	ACATTGCAAATAGGGGTATG	177	JOE

[a] 6-Carboxyfluorescein (6-FAM); 4,5-Dichloro-dimethoxy-fluorescein (JOE).

Supplementary figure legend

Figure S1. Venn diagram showing the number and relationship of proteins differentially expressed for the light intensity and temperature comparisons from the *C. formosensis*. PPFD, photosynthetic photon flux density or light intensity (μmol m^{-2} s^{-1}).

Figure S2. Venn diagram showing the number and relationship of proteins differentially expressed for the light intensity and temperature comparisons from the *C. obtusa* var. *formosana*. PPFD, photosynthetic photon flux density or light intensity (μmol m^{-2} s^{-1}).

Figure S3. Genetic structure of populations of (a) *C. formosensis*, and (b) *C. obtusa* var. *formosana* saplings.

Figure S4. Cluster analysis of the constituents of volatile compounds from (a) *C. formosensis*, and (b) *C. obtusa* var. *formosana* saplings using static-headspace.

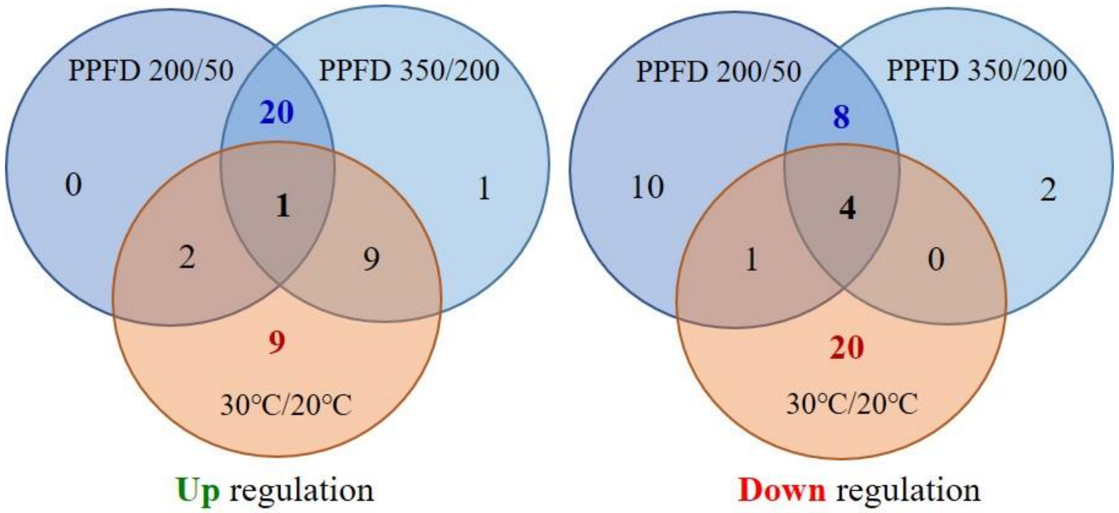

Figure S1. Venn diagram showing the number and relationship of proteins differentially expressed for the light intensity and temperature comparisons from the *C. formosensis*. PPFD, photosynthetic photon flux density or light intensity (μmol m^{-2} s^{-1}).

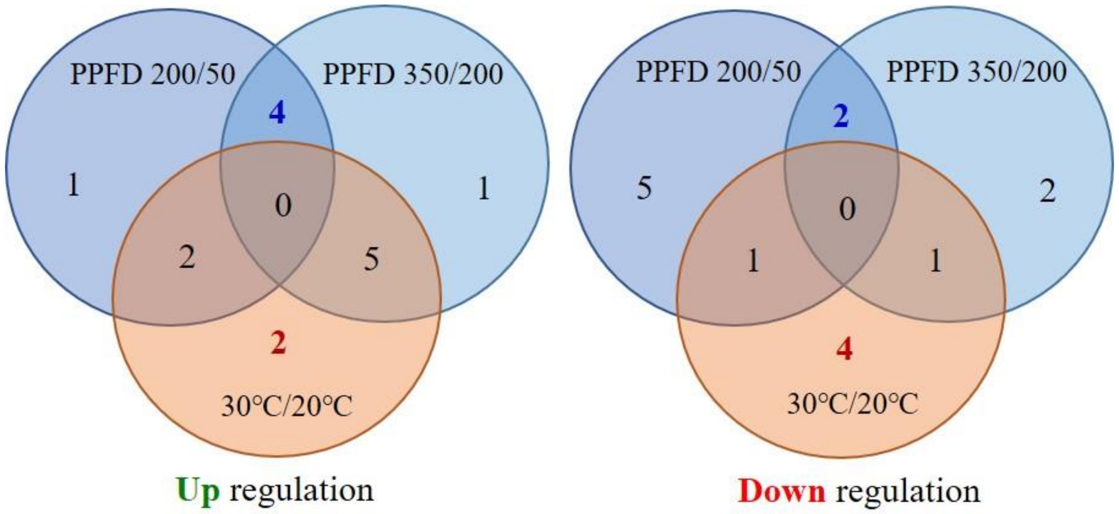

Figure S2. Venn diagram showing the number and relationship of proteins differentially expressed for the light intensity and temperature comparisons from the *C. obtusa* var. *formosana*. PPFD, photosynthetic photon flux density or light intensity (μmol m^{-2} s^{-1}).

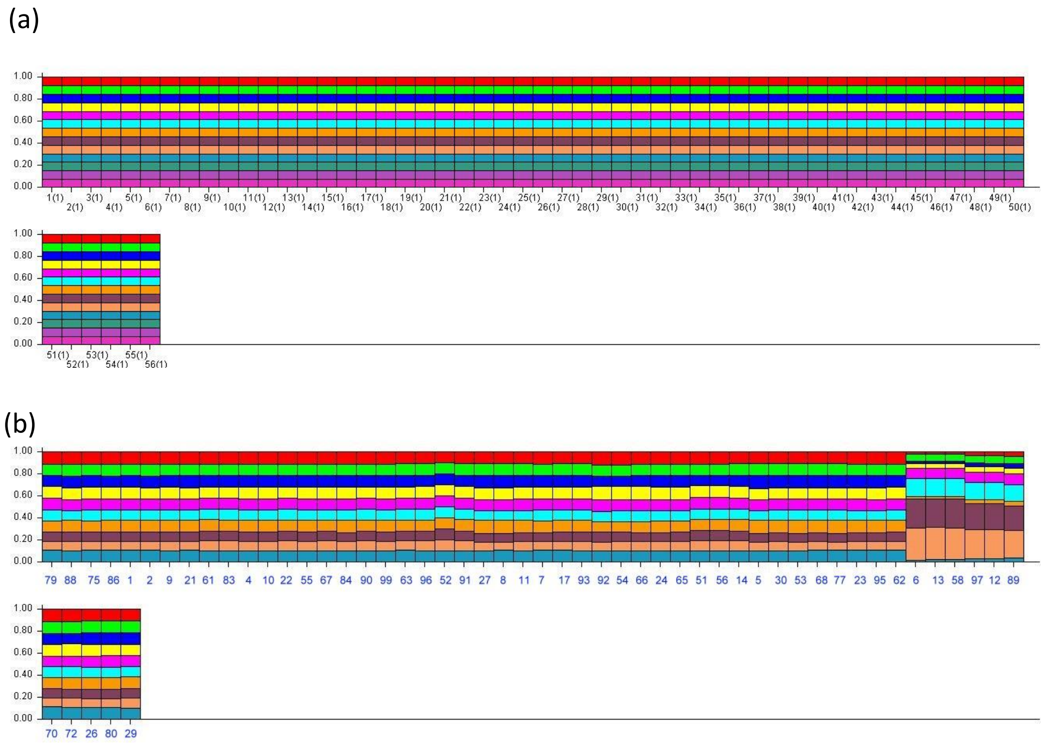

Figure S3. Genetic structure of populations of (a) *C. formosensis*, and (b) *C. obtusa* var. *formosana* saplings.

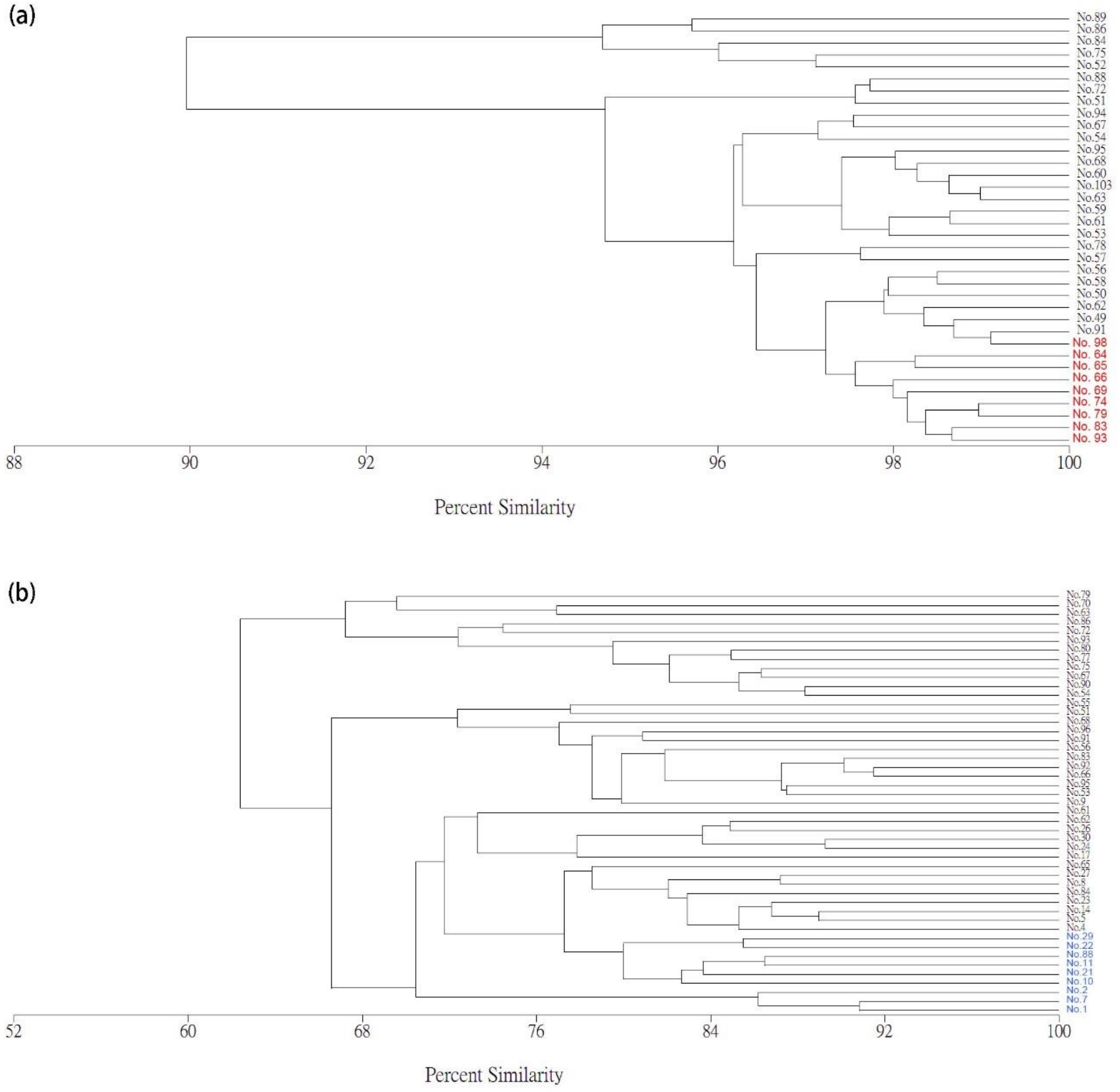

Figure S4. Cluster analysis of the constituents of volatile compounds from (a) *C. formosensis*, and (b) *C. obtusa* var. *formosana* saplings using static-headspace.
